# CLOCKWORK ORANGE Enhances PERIOD Mediated Rhythms in Transcriptional Repression by Antagonizing E-box Binding by CLOCK-CYCLE

**DOI:** 10.1371/journal.pgen.1006430

**Published:** 2016-11-04

**Authors:** Jian Zhou, Wangjie Yu, Paul E. Hardin

**Affiliations:** 1 Department of Biology, Texas A&M University, College Station, Texas, United States of America; 2 Center for Biological Clocks Research, Texas A&M University, College Station, Texas, United States of America; Washington University in Saint Louis School of Medicine, UNITED STATES

## Abstract

The *Drosophila* circadian oscillator controls daily rhythms in physiology, metabolism and behavior via transcriptional feedback loops. CLOCK-CYCLE (CLK-CYC) heterodimers initiate feedback loop function by binding E-box elements to activate *per* and *tim* transcription. PER-TIM heterodimers then accumulate, bind CLK-CYC to inhibit transcription, and are ultimately degraded to enable the next round of transcription. The timing of transcriptional events in this feedback loop coincide with, and are controlled by, rhythms in CLK-CYC binding to E-boxes. PER rhythmically binds CLK-CYC to initiate transcriptional repression, and subsequently promotes the removal of CLK-CYC from E-boxes. However, little is known about the mechanism by which CLK-CYC is removed from DNA. Previous studies demonstrated that the transcription repressor CLOCKWORK ORANGE (CWO) contributes to core feedback loop function by repressing *per* and *tim* transcription in cultured S2 cells and in flies. Here we show that CWO rhythmically binds E-boxes upstream of core clock genes in a reciprocal manner to CLK, thereby promoting PER-dependent removal of CLK-CYC from E-boxes, and maintaining repression until PER is degraded and CLK-CYC displaces CWO from E-boxes to initiate transcription. These results suggest a model in which CWO co-represses CLK-CYC transcriptional activity in conjunction with PER by competing for E-box binding once CLK-CYC-PER complexes have formed. Given that CWO orthologs DEC1 and DEC2 also target E-boxes bound by CLOCK-BMAL1, a similar mechanism may operate in the mammalian clock.

## Author Summary

Circadian clocks control daily rhythms in animal, plant and fungal physiology, metabolism and behavior via transcriptional feedback loops. In *Drosophila*, the CLOCK-CYCLE (CLK-CYC) activator complex binds E-box regulatory sequences to initiate transcription of hundreds of effector genes including their own repressors, PERIOD (PER) and TIMELESS (TIM), which feed back to repress CLK-CYC until they are degraded, thus allowing another cycle of CLK-CYC activation. Although the repression process is critical for the stability and accuracy of circadian timekeeping, how PER-TIM complexes maintain a transcriptionally repressed state for many hours is not well understood. Here we demonstrate that the transcription factor CLOCKWORK ORANGE (CWO) antagonizes CLK-CYC E-box binding, thus enhancing the removal of CLK-CYC from E-boxes to maintain transcriptional repression. This process requires PER, which suggests that PER-TIM and CWO cooperate to maintain a transcriptionally repressed state by removing CLK-CYC from E-boxes. These results demonstrate that PER-TIM requires CWO to effectively repress circadian transcription, and given that circadian transcriptional regulators are well conserved, this mechanism may function to repress transcription in other animals including humans.

## Introduction

Almost all organisms from *Cyanobacteria* to humans have internal circadian clocks that drive daily rhythms in physiology, metabolism and behavior, thereby synchronizing internal processes with the external environment. In eukaryotes, the circadian clock keeps time via one or more transcriptional feedback loops [1]. In *Drosophila*, a heterodimer formed by CLOCK (CLK) and CYCLE (CYC) binds E-box sequence activates transcription to initiate clock function. In the core loop, CLK-CYC activates *period* (*per*) and *timeless* (*tim*) transcription during mid-day, effecting a rise in *per* and *tim* mRNA levels that peaks during the early evening. PER and TIM proteins then accumulate, form a dimer, and move into the nucleus to bind CLK-CYC during the night, thereby inhibiting their transcriptional activity until PER and TIM are degraded early in the morning [2,3]. Another interlocked transcriptional feedback loop is also regulated by the core feedback loop. In this loop, CLK-CYC activates transcription of *vrille* (*vri*) and *PAR-domain protein 1ɛ* (*Pdp1ɛ*), which bind D-boxes to repress and activate transcription, respectively, and drive RNA cycling of *Clk* and other output genes in the opposite phase as *per*, *tim*, *vri* and *Pdp1ɛ* [4–6].

PER was previously found inhibit CLK-CYC binding to E-boxes *in vitro* [7], which suggests that the rhythmic transcription of CLK target genes are mediated by PER-dependent rhythms in E-box binding by CLK-CYC. Chromatin immunoprecipitation (ChIP) experiments using fly heads support this model, showing that CLK-CYC rhythmically bind E-boxes in the *per* circadian regulatory sequence (CRS) and the *tim* upstream sequence [8]. However, the mechanism by which CLK-CYC heterodimers are removed from E-boxes during repression is not well understood. PER is required for the rhythmic binding of CLK complexes, as CLK constantly binds to *per* and *tim* promoters in *per*^*01*^ flies [8], indicating that PER inhibits transcription by removing CLK-CYC from E-boxes. Interestingly, co-expression of another transcription factor, CLOCKWORK ORANGE (CWO), strongly enhanced PER-mediated repression in cultured *Drosophila* Schneider 2 (S2) cells [9], suggesting that PER is unable to efficiently remove CLK from DNA in the absence of other transcription repressors.

Previous studies demonstrated that CWO, a basic helix-loop-helix (bHLH)-ORANGE transcriptional factor [10], is a direct target of CLK-CYC [9,11,12]. In *Drosophila* Schneider 2 (S2) cells, overexpression of CWO reduces the basal transcription of *per*, *tim*, *vri* and *Pdp1ɛ* promoter-driven luciferase reporter genes [9,12,13]. Furthermore, in the presence of PER, CWO repress CLK mediated transcription 5–10 fold in S2 cells, indicating that CWO is a strong transcription repressor that can cooperate with PER to repress CLK-CYC mediated transcription [9]. In *cwo* mutants or *cwo* RNAi knockdown flies, the levels of *per*, *tim*, *vri* and *Pdp1ɛ* mRNAs are increased during the early to mid-morning [9,12]. These results suggest that CWO co-represses CLK-CYC activity along with PER during the end of a cycle [9,12]. However, the mechanism through which CWO represses CLK-CYC mediated gene transcription remains unknown.

In this study we demonstrate that CWO and CLK bind core clock gene E-boxes in a reciprocal pattern across the circadian cycle *in vivo*, which suggests that CWO competes with CLK to bind E-boxes. We also show that CWO acts to decrease CLK binding to *tim* E-boxes during early morning, when PER binds CLK-CYC to reduce its binding to DNA [8], but not during early night when CLK-CYC strongly binds E-boxes in the absence of PER. These results suggest a model for CWO function where CWO has low DNA binding affinity compared to CLK-CYC complexes during the activation phase, but has higher affinity compared to CLK-CYC-PER complexes, and is thus capable of removing CLK-CYC-PER complexes from E-boxes to consolidate and maintain repression. Constant high CWO binding to the *tim* promoter in *Clk*^*out*^ flies (i.e. comparable to binding at ZT2 in wild-type) and constant low CWO binding in *per*^*01*^ flies (i.e. comparable to binding at ZT14 in wild-type) supports our model for CWO repression. As a whole, these results suggest that CWO co-represses CLK-CYC activity with PER by competing with CLK-CYC-PER complexes for E-box binding, therefore promoting the transition to off-DNA repression.

## Results

### CWO is present at constant levels and rhythmically binds E-boxes in a reciprocal pattern compared to CLK

**Fig 1:**
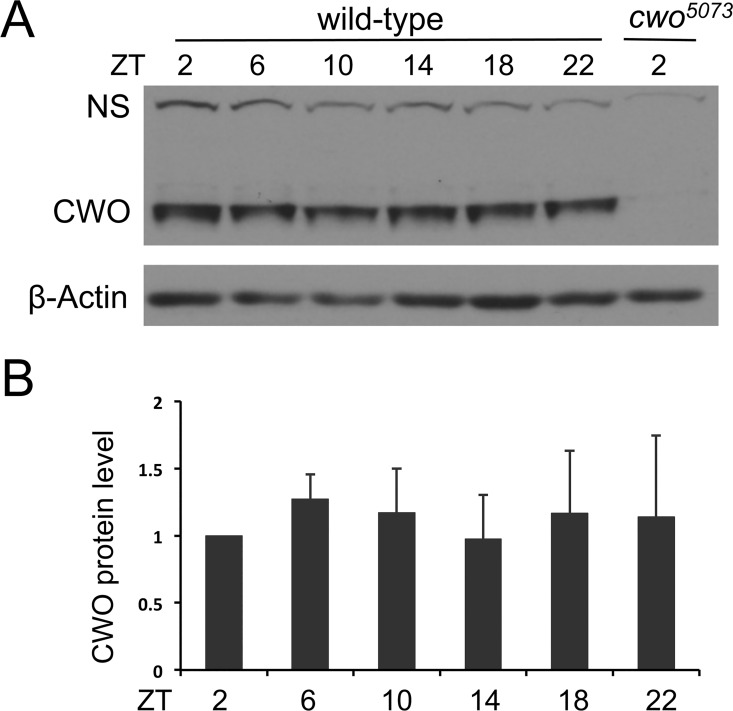
CWO protein is present at constant levels in fly heads. **(A)** Western blot of head extracts from wild-type and *cwo*^*5073*^ flies collected at the indicated times were probed with CWO antiserum. β-Actin or a nonspecific band (NS) were used as loading controls. **(B)** Quantification of CWO levels in the blot from panel A and two additional western blots containing samples from independent collections. Error bars indicate the SD (n = 3).

Earlier studies demonstrated that *cwo* mRNA cycles in phase with *per*, *tim*, *vri*, and *Pdp1*, but with a higher basal level, and thus lower amplitude [9,12–14]. To determine whether CWO protein levels also cycle, western analysis was carried out using head extracts from wild-type flies collected every 4 hours in a 12-h light/12-h dark (LD) cycle. We find that the levels of CWO do not change throughout an LD cycle (Fig 1), consistent with previous results [15]. Given that *cwo* mRNA levels cycle, it is possible that constant CWO levels result from post-transcriptional regulation or a long half-life.

**Fig 2:**
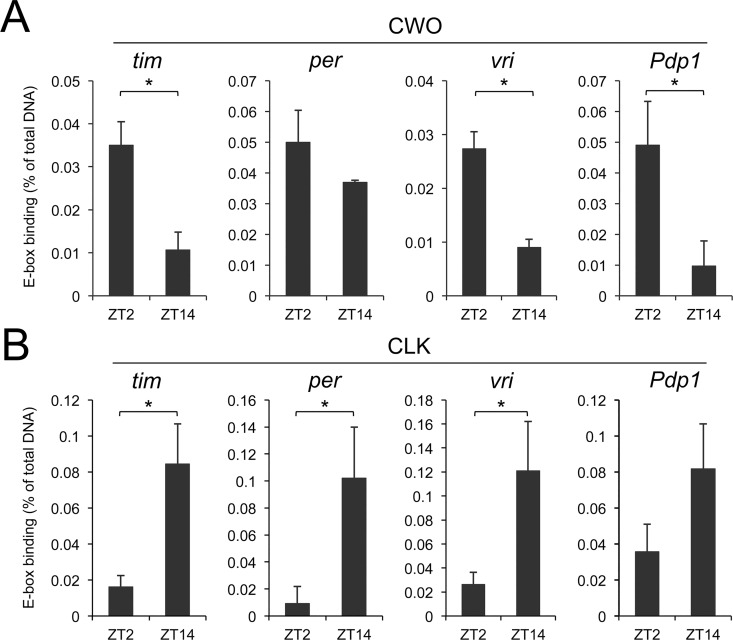
CWO rhythmically binds E-boxes of core clock genes in antiphase to CLK. **(A)** ChIP assays were performed on wild-type flies collected at ZT2 and ZT14. The relative level of CWO binding to *tim*, *per*, *vri* and *pdp1* E-boxes was determined by qPCR analysis of samples immunoprecipitated with CWO antiserum (see Materials and Methods). The mean values of three independent ChIP assays were calculated and plotted. Error bars indicate the SEM (n = 3, *p<0.05, Student’s t-test). **(B)** ChIP assays of samples immunoprecipitated with CLK antiserum were performed, quantified and plotted as described for panel A. Error bars indicate the SEM (n = 3, *p<0.05, Student’s t-test).

CWO contains a bHLH domain, a structural motif that characterizes a family of E-box binding transcription factors [16–19], which suggests that CWO may regulate CLK-CYC target gene transcription via E-box binding. Previous ChIP-on-chip and gel-shift analyses in S2 cells demonstrated that CWO specifically binds to the E-box of core clock genes [12,13], however it is still unknown whether CWO binds those core clock genes *in vivo*, and whether the binding intensity changes throughout the day. To test these possibilities, ChIP assays were carried out on wild-type flies collected in the early morning (ZT2) and in the early night (ZT14) using CWO and CLK antisera. Fragments containing upstream E-boxes from *tim*, *per*, *Pdp1* and *vri*, which are necessary for high-amplitude mRNA cycling *in vitro* or *in vivo* [4,5,20–25], were amplified from the immunoprecipitates and then quantified. In CWO immunoprecipitates, the *tim*, *vri* and *Pdp1* E-box containing fragments were two to threefold more abundant at ZT2 than at ZT14 (Fig 2A), suggesting that CWO binding is time-dependent, though the dynamic binding of CWO on the *per* E-box fragment is less robust than the others. Importantly, this temporal binding pattern is antiphase to CLK binding, as CLK shows high binding intensity during the night at ZT14 and low binding during the daytime at ZT2 (Fig 2B), consistent with previous results [8,11].

### CWO and CLK bind tandem E-boxes upstream of *tim*

**Fig 3:**
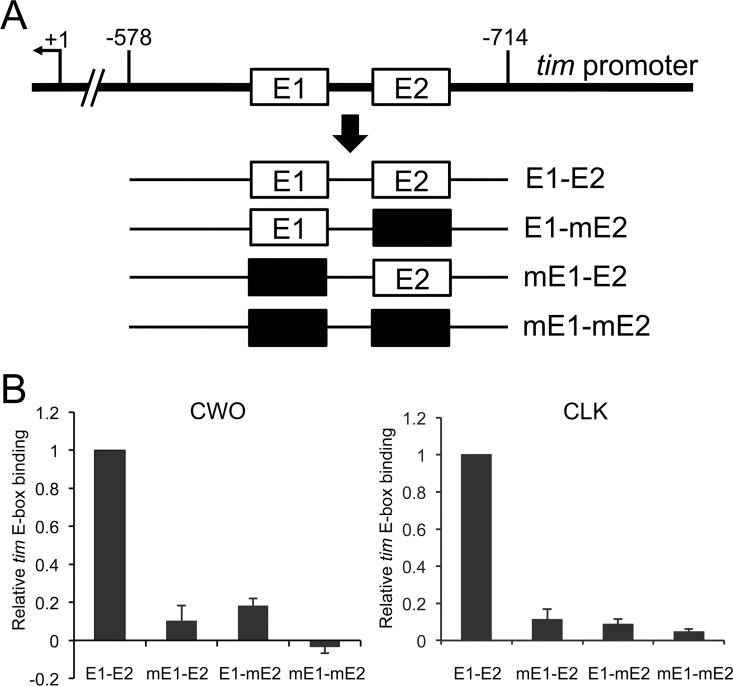
CWO and CLK bind the same tandem E-boxes in the *tim* circadian enhancer. **(A)** Schematic diagram of wild-type and mutant *tim* circadian enhancer transgenes. A 136bp *tim* circadian enhancer fragment that extends from -578 to -714 relative to the *tim* transcription start (+1; see [24]) was used to generate transgenes with wild-type or mutant combinations of tandem E-box 1 (E1) and E-box 2 (iE2) motifs. Wild-type and mutant *tim* circadian enhancer fragments were generated via PCR, cloned into the pHPdesteGFP reporter plasmid, and used to generate transgenic flies via PhiC31 recombination (see Materials and Methods). The resulting *tim* circadian enhancer transgenes contain wild-type E1 and E2 (E1-E2), mutant E1 and wild-type E2 (mE1-E2), mutant E2 and wild-type E1 (E1-mE2), or mutant E1 and E2 (mE1-mE2) E-boxes. Black boxes, mutant E1 or E2 E-boxes; double backslash, virtual break in the *tim* promoter sequence. **(B)** ChIP assays on flies containing the E1-E2, mE1-E2, E1-mE2 or mE1-mE2 *tim* circadian enhancer transgenes. The relative level of CWO binding at ZT2 or CLK binding at ZT14 was determined as described in Fig 2A. Relative binding of CWO or CLK was normalized to the maximum E1-E2 value of 1.0, then the means of each data set were calculated and plotted. Error bars represent the SEM (n = 3).

The reciprocal binding pattern of CLK and CWO implies that these transcription factors compete for E-box binding. If so, both CLK and CWO must occupy the same E-boxes. To test this possibility, we determined how mutating E-boxes upstream of *tim* affected CLK and CWO binding. The circadian enhancer upstream of *tim* is comprised of two tandem E-boxes that are spaced seven nucleotides apart [24,26], a structure that is conserved among core clock genes in various species [27]. Both of these E-boxes were indispensable for *tim* mRNA expression in S2 cells [24], suggesting that these tandem E-box motifs are binding sites for both CLK and CWO. To determine if this is the case, a series of 136bp fragments from the *tim* promoter containing an E-box1 (E1) mutant (mE1-E2), an E-box 2 (E2) mutant (E1-mE2), an E1 and E2 double mutant (mE1-mE2) or a control with wild-type E-boxes (E1-E2) were generated, inserted into the pHPdestGFP vector [28], and targeted to the attP18 genomic site (Fig 3A).

To confirm that this promoter fragment is sufficient to drive rhythmic expression, we carried out quantitative reverse transcription-PCR (qRT-PCR) to monitor *GFP* mRNA levels in flies collected every 4-h during an LD cycle. Quantification of *GFP* mRNA levels in flies with WT *tim* promoter shows a ~10-fold diurnal rhythm with a peak at ZT14 and a trough at ZT2 to ZT6 (S1 Fig), consistent with timing and amplitude of *per* and *tim* mRNA cycling in wild-type flies [29,30]. However, even at the normal *tim* mRNA peak (ZT14), mE1-E2, E1-mE2 and mE1-mE2 flies express little or no eGFP mRNA (S1 Fig), indicating that both E1 and E2 are indispensable for expression of *tim* mRNA *in vivo*. This result is consistent with previous *tim*-luciferase reporter results in S2 cells [24].

We next carried out ChIP assays using CWO and CLK antisera on the same fly strains to test whether E1 and E2 are required for CWO and CLK binding. At ZT2, when CWO strongly binds to the *tim* promoter, CWO binding intensity was drastically reduced in mE1-E2, E1-mE2 and mE1-mE2 flies compared to WT (Fig 3B). Likewise, CLK binding intensity was drastically reduced in mE1-E2, E1-mE2 and mE1-mE2 flies compared to WT at ZT14, when CLK binding is strongest (Fig 3B). These results indicate that both E1 and E2 are indispensable for both CWO and CLK binding to the *tim* circadian enhancer. Given that CWO specifically targets E-boxes in S2 cells by Gel-shift analyses [13], we conclude that both CLK and CWO bind intact tandem E1-E2 motifs *in vivo*. In mice, CLK-BMAL1 dimers cooperatively bind tandem E-boxes *in vitro* [27,31], and this may be the case for CWO given the requirement for both E1 and E2 E-boxes.

### CWO represses CLK binding to *tim* promoter during transcription repression

**Fig 4:**
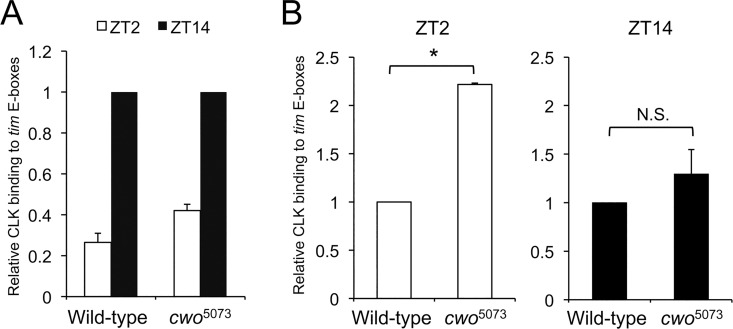
CWO reduces CLK binding to *tim* E-boxes during transcriptional repression. ChIP assays were performed on wild-type and *cwo*^*5073*^ flies collected at ZT2 and ZT14, and the relative level of CLK binding to *tim* E-box-containing fragments was determined as described in Fig 2A. (A) CLK binding signal was normalized to a ZT14 value of 1.0 for wild-type and *cwo*^*5073*^ flies, respectively, and the mean values at ZT2 from each data set (n = 3) were calculated and plotted. (B) CLK binding signal from *cwo*^*5073*^ flies was normalized to a wild-type value of 1.0 at ZT2 and ZT14, respectively, and the mean values of each data set were calculated and plotted. Error bars represent the SEM (n = 3, *p<0.05, Student’s t-test).

Previous studies showed that increasing the level of CWO expression reduces *per*, *tim*, *vri* and *Pdp1ɛ* mRNA levels in S2 cells and that their trough mRNA levels are higher in *cwo* mutant or knockdown flies, indicating that CWO acts to repress CLK-mediated gene transcription *in vitro* and *in vivo* [9,12–14]. Given that CWO and CLK bind to the same E-box motif, we wondered whether CWO represses CLK-mediated transcription by inhibiting CLK binding. To test this possibility, ChIP assays were carried out using CLK antiserum on wild-type and *cwo*^*5703*^ flies at the trough (ZT2) and peak (ZT14) times of CLK-CYC target gene transcription and mRNA abundance in LD. Although *cwo*^*5703*^ mutants lengthen the period of activity rhythms by 2–3h in DD [9,13], the peak and trough phases of CLK-CYC target gene transcription and mRNA abundance are comparable in *cwo*^*5703*^ mutants and wild-type flies in LD [9,13]. We find that CLK binds *tim* E-boxes with a robust rhythm in wild-type flies and a lower amplitude rhythm in the *cwo*^*5703*^ mutant (Fig 4A). However, the intensity of CLK binding in *cwo*^*5703*^ is significantly increased at ZT2 compared to wild-type, indicating that CWO acts to reduce CLK-CYC binding at the trough of its binding cycle (Fig 4B). Given that CWO strongly binds *tim* E-boxes at ZT2 (Fig 2A), we propose that CWO inhibits CLK-CYC binding during the repression phase by antagonizing PER-CLK-CYC complexes to maintain off-DNA repression. There was no significant difference in CLK binding between *cwo*^*5703*^ and wild-type at ZT14 (Fig 4B), despite decreased peak levels of *per*, *tim*, *vri* and *Pdp1ɛ* mRNA at ZT14 in *cwo* mutant and RNAi knockdown flies [9,12–14], suggesting that CWO has little impact on CLK-CYC binding in the absence of PER.

### PER is required for CWO to displace CLK-CYC binding on E-boxes

**Fig 5:**
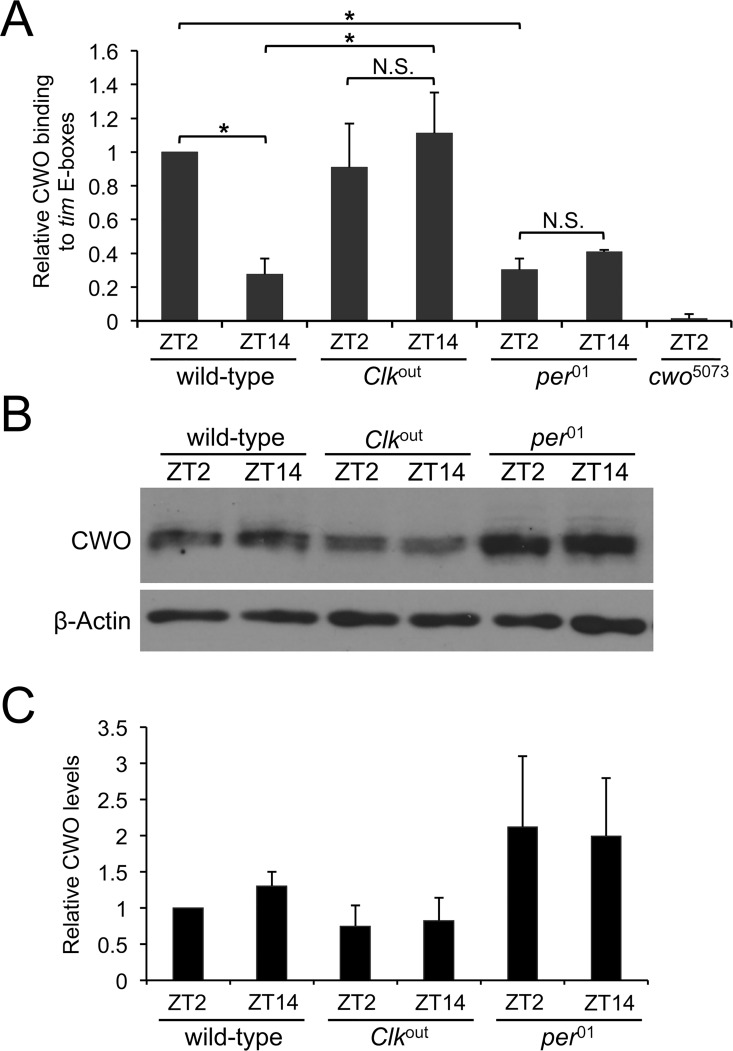
PER is required for CWO to compete with CLK-CYC for E-box binding. **(A)** ChIP assays were performed on wild-type, *Clk*^*out*^ and *per*^*01*^ flies collected at ZT2 and ZT14, and *cwo*^*5073*^ flies collected at ZT2, as described in Fig 2A. The relative level of CWO binding to *tim* E-box-containing fragments was determined as described in Fig 2A. The signal from each sample was normalized to the wild-type ZT2 value of 1.0, then the means of each data set (n = 3) were calculated and plotted. Error bars represent the SEM (n = 3, *significantly different, ANOVA followed by Student-Newman-Keuls post-hoc test). **(B)** Western blot of head extracts from the same genotypes shown in panel A were probed with CWO antiserum. **(C)** Quantification of CWO levels in the blot from panel B and two additional Western blots containing samples from independent collections. Error bars indicate the SD.

Given that CWO suppresses CLK binding at ZT2 in the early morning but not at ZT14 during the early evening (Fig 4B), it is possible that PER is necessary for CWO to antagonize CLK E-box binding since PER accumulates to high levels in the nucleus around dawn and is at low levels in the cytoplasm around dusk [32]. Indeed, our results support a model developed previously to explain cooperation between CWO and PER to repress CLK-CYC mediated transcription in S2 cells [9]. In this model, CWO is proposed to compete with CLK-CYC heterodimers for E-box binding only when PER binds CLK-CYC, thereby reducing their affinity for E-box binding. To test this model, we performed ChIP assays using CWO antiserum on wild-type, *Clk*^*out*^ and *per*^*01*^ flies collected at ZT2 and ZT14 in LD. In *Clk*^*out*^ flies, which necessarily lack CLK-CYC heterodimers [33], CWO is bound to *tim* E-boxes at both ZT2 and ZT14 with binding signals comparable to the strong CWO binding in wild-type flies at ZT14 (Fig 5A). In contrast, in *per*^*01*^ flies, which lack PER-dependent repression of CLK-CYC activation [34], low binding signals of CWO were detected at ZT2 and ZT14, indicating that PER is indeed required for CWO to bind E-boxes (Fig 5A). Moreover, CWO binding was significantly increased in *Clk*^*out*^ versus wild-type flies at ZT14, indicating that CLK-CYC binding at ZT14 reduces CWO binding. Likewise, a significant increase in CWO binding was also seen in wild-type versus *per*^*01*^ flies at ZT2, indicating that PER enhances CWO binding (Fig 5A).

**Fig 6:**
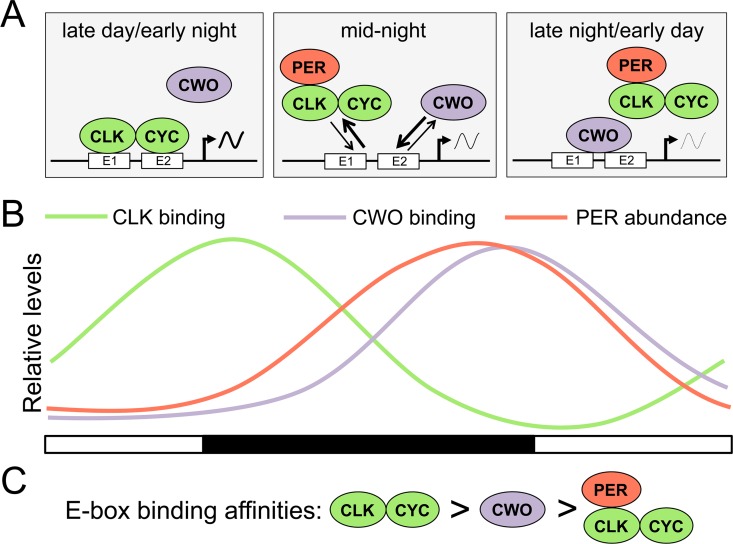
Model for PER dependent binding competition between CWO and CLK-CYC on E-boxes. **(A)** Diagrams depicting clock protein interactions and E-box binding at different times of day (gray rectangles). During late day/early night, CLK-CYC (green ovals) initiate the transcription cycle by binding to E-boxes (white rectangle) in the presence of CWO (purple oval), which is unable to bind E-boxes. During mid-night, PER (red oval) enters the nucleus and interacts with CLK-CYC, producing PER-CLK-CYC complexes that allow CWO binding to E-boxes. During late night/early day, the high PER levels insure efficient PER-CLK-CYC complex formation, thus allowing strong CWO binding to E-boxes. **(B)** Graph showing the relative levels of CLK-CYC binding (green line), CWO binding (purple line) and PER abundance (red line) during a light (white rectangles) and dark (black rectangles) cycles. **(C)** Proposed E-box binding affinities, where CLK-CYC binding is greater than (>) CWO, and CWO binding is greater than PER-CLK-CYC.

To determine whether differences in CWO binding in *Clk*^*out*^ and *per*^*01*^ flies were due to differences in CWO protein levels, we carried out western analysis using head extracts from these mutants collected at ZT2 and ZT14. Since *cwo* transcription is regulated in part by the transcriptional feedback loop, CWO protein levels are slightly lower in *Clk*^*out*^ flies and slightly higher in *per*^*01*^ flies (Fig 5B and 5C). However, the lower levels of CWO in *Clk*^*out*^ resulted in higher E-box binding, and higher CWO protein levels in *per*^*01*^ resulted in lower E-box binding. This result suggests that the differences in CWO-E-box binding are not due to altered CWO protein levels, but due to the relative DNA binding affinities of CWO and CLK in these mutants. These results, taken together, strongly support and extend the model described by Kadener et al., 2007, for CWO binding as it relates to CLK-CYC repression. When CLK-CYC targets are activated, CLK-CYC binds DNA with higher affinity than CWO, thus CLK binding is not altered in the presence or absence of CWO. When CLK-CYC targets are repressed, PER binds CLK-CYC complexes and decreases their DNA binding affinity, thereby favoring CWO binding to E-boxes and enhancing PER mediated removal of CLK-CYC-PER complexes from the DNA (Fig 6). Although we can’t exclude the possibility that PER enables CWO E-box binding independent of its interaction with CLK-CYC, the available evidence strongly supports the model proposed.

## Discussion

Rhythmic binding of CLK-CYC to E-boxes is essential for rhythmic transcription of the core circadian oscillator genes *per* and *tim* in *Drosophila*. CLK-CYC bind E-boxes upstream of *per* and *tim* in the late day and early night to activate transcription; and is released from these binding sites during late night [8,35,36]. Previous work demonstrated that CLK constitutively binds *per* and *tim* E-boxes in *per*^*01*^ flies, indicating that PER is essential for rhythmic binding of CLK-CYC, and is key to removing CLK-CYC from E-boxes [8]. In this study we report that CWO also contributes to removing CLK-CYC from E-boxes. In *cwo*^*5703*^ mutant flies, CLK binding intensity is significantly increased at the trough of its binding cycle, suggesting that repression is incomplete in the absence of CWO (Fig 4).

We find that CWO and CLK bind E-boxes upstream of *tim* in a reciprocal manner during a daily cycle, and that CLK shows significantly increased binding intensity at the trough of its binding cycle in *cwo* mutant flies, indicating that CWO acts to antagonize CLK-CYC binding. Given that both CWO and CLK are constitutively expressed (Fig 1; [8]), we believe that the key driver for the transition between dynamic CLK-CYC and CWO binding is the accumulation of PER, which alters the relative affinity of E-box binding by CLK-CYC. CWO shows low levels of *tim* E-box binding in *per*^*01*^ flies, in which CLK-CYC constantly bind E-boxes, but shows high levels of *tim* E-box binding in *Clk*^*out*^ flies that lack CLK expression and E-box occupancy. These results suggest that CWO E-box binding affinity is lower than the CLK-CYC heterodimer and higher than the CLK-CYC-PER complex, which could account for the PER-dependent rhythms in CLK-CYC and CWO binding (Fig 6). During late day and early night, CLK-CYC binds E-boxes to activate transcription in the presence of CWO because CLK-CYC has higher DNA binding affinity. PER starts to accumulate in the nucleus during the night and interacts with CLK-CYC, which decreases CLK-CYC DNA interaction via reduced DNA binding affinity. Consequently, CWO displaces CLK-CYC-PER from E-boxes by binding with comparatively higher affinity. Once CLK-CYC-PER is removed, CWO occupancy on E-boxes prohibits CLK-CYC-PER from re-binding, thus maintaining transcriptional repression (Fig 6).

Unlike the constitutive CLK-CYC E-box binding in *per*^*01*^ flies [8], CLK-CYC binding is rhythmic in *cwo*^*5703*^ flies, but with a dampened amplitude due to elevated CLK binding at the trough (Fig 4A). This low amplitude rhythm in CLK binding may explain why a large proportion of *cwo*^*5703*^ flies show long period rhythms rather than losing rhythmicity entirely like *per*^*01*^ mutants [9,12–14]. We speculate that the long period phenotype is caused in part by a prolonged repression process. Based on the current model for repression of CLK-CYC transcription, PER-TIM complexes first bind CLK-CYC, thereby removing CLK-CYC from the E-boxes and inhibiting *per* and *tim* transcription, then PER and TIM degradation enables CLK-CYC binding to start another cycle of transcription [3]. Both of these steps could be delayed in a *cwo* mutant. In the absence of CWO it takes longer to remove CLK-CYC from the DNA; PER alone can repress CLK-CYC binding to some degree, but CLK-CYC-PER complexes still weakly bind E-boxes if CWO is absent, thus reducing CLK-CYC repression compared to wild-type flies. The outcome of incomplete repression of CLK-CYC E-box binding would be an increase in the trough levels of *per* and *tim* mRNAs, which is exactly what was observed in *cwo* mutant and RNAi knockdown strains [9,12–14]. Higher *per* and *tim* mRNA levels would in turn increase PER and TIM expression during the repression phase [14]. Higher levels of PER and TIM would not repress CLK-CYC binding efficiently in the absence of CWO, but would take longer to be degraded, thereby delaying the next cycle of transcriptional activation.

In addition to the increased trough levels of core clock gene mRNAs in *cwo* mutant and RNAi knockdown flies, the peak levels of these mRNAs are lower, particularly during DD [9,12–14]. Decreasing *per* mRNA levels also lengthen circadian period [37], thus making it difficult to determine the extent to which a lower mRNA peak or increased mRNA trough contributes to period lengthening in *cwo* mutant and RNAi knockdown flies. CLK binding at the peak of transcription is not significantly lower in *cwo*^5073^ than wild-type during LD (Fig 4B), which argues that CWO enhances CLK-CYC transcriptional activity independent of CLK-CYC E-box binding. Additional experiments will be needed to decipher the mechanism underlying this CWO dependent increase in CLK-CYC transcription.

PER dependent repression of CLK-CYC transcription is thought to occur in two stages. First, PER is recruited to circadian promoters by interacting with CLK to form PER-CLK-CYC complexes “on-DNA”, which inhibit CLK-CYC dependent transcription via an unknown mechanism. Subsequently, a decrease in the DNA binding affinity of PER-CLK-CYC complexes results in their release from DNA to initiate ‘‘off-DNA” phase of repression [35]. According to our model, CWO is critical for the transition to, and maintenance of, off-DNA repression. When PER-CLK-CYC complexes with low DNA affinity are formed, CWO promotes off-DNA repression by competing with CLK-CYC-PER complex for E-box binding. CWO occupancy on E-boxes then prevents PER-CLK-CYC from re-binding, thereby maintaining off-DNA repression.

In mammals, a similar pattern of antagonistic binding on E-boxes between transcription factors was recently reported; USF1 and a mutant form of CLOCK, CLOCK^Δ19^, bind to the same tandem E-boxes in a reciprocal manner. Wild-type CLOCK-BMAL1 complex binds E-boxes with much higher affinity than USF1, but CLOCK^Δ19^-BMAL1 binds E-boxes with a similar affinity to USF1, thus allowing USF1 to bind E-boxes [31]. Although this competitive binding is not thought to impact feedback loop function under normal circumstances, it demonstrates that other transcription factors can out-compete CLOCK-BMAL1 for E-box binding if the DNA binding affinity of CLOCK-BMAL1 is reduced. In this case CLOCK-BMAL1 binding is compromised by the *Clock*^Δ19^ mutation, but other mechanisms such as interactions with repressors and protein modifications could also reduce the binding affinity of CLOCK-BMAL1 or its orthologs.

As in Drosophila, rhythmic binding of CLOCK-BMAL1 to E-boxes drives circadian transcription in mammals (reviewed in [38]). Recent ChIP-seq analyses in mouse liver revealed time-dependent binding of CLOCK, BMAL1 and key negative feedback components including PER1, PER2, CRY1 and CRY2 [27,39–41]. The mechanism underlying the dynamic DNA occupancy of these transcription factors is not known, but previous work shows that the PER2-CLOCK interaction is required to initiate repression of CLOCK-BMAL1 dependent transcription [42], which suggests that CLOCK-BMAL1 may be removed from E-boxes by the same mechanism as CLK-CYC in Drosophila. A recent genome-wide nucleosome analysis in mouse liver revealed that rhythmic E-box binding by CLOCK-BMAL1 removes nucleosomes [43]. However, despite rhythmic CLOCK-BMAL1 binding, nucleosome occupancy on E-boxes is always well below surrounding sequences, even in Bmal1^-/-^ mutant livers [43]. This result indicates that chromatin at CLOCK-BMAL1 target sites is not closed even when there is no CLOCK-BMAL1 binding, suggesting that other transcription factors may occupy these E-boxes when CLOCK-BMAL1 is absent. These results, taken together, suggest that rhythms in activator binding may be controlled by a common mechanism in *Drosophila* and mammals.

The mammalian orthologs of CWO, called DEC1 and DEC2 (and also SHARP2 and SHARP1, respectively), suppress CLOCK-BMAL1-induced activation [44–50]. Gel mobility shift and ChIP assays *in vitro* revealed that both DEC1 and DEC2 bind to E-box motifs targeted by CLK-BMAL1 [45–49], and the DNA-binding domain is required for DEC1 to regulate CLK-BMAL1-induced transactivation [48]. In addition, DEC1/2 shows synergistic activity to PER1 in the regulation of clock gene mRNA levels in the SCN, as exemplified by significant changes in the period of circadian activity rhythms when null mutants for *Dec1*, *Dec2* or both *Dec1* and *Dec2* are combined with that for *Per1* [44]. In contrast to the constant levels of CWO, DEC1 protein is rhythmically expressed in mouse liver, where DEC1 levels are high when PER-CRY complexes repress CLK-BLMAL1 transcription [51]. Taken together, these results raise the possibility that DEC1 and DEC2 may be a functional counterpart of CWO in competing with CLOCK-BMAL1 for E-box binding to repress CLOCK-BMAL1-mediated transcription.

## Materials and Methods

### Transgene construction and transgenic fly generation

DNA fragments containing wild-type or mutant E-boxes from the upstream *tim* circadian enhancer were used to construct GFP-reporter transgenes. These 136bp fragments extend from -578 to -714 relative to the *tim* transcription start site, and contain “E1-E2” E-box motifs that are wild-type (E1-E2), E1 mutant (mE1-E2), E2 mutant (E1-mE2) or E1-E2 mutant (mE1-mE2). These wild-type and mutant E-box fragments were generated by PCR amplification using the following primer sets: E1-E2, 5’-CACCTTTGGCAAATAAACGTGCGGCA-3’ and 5’-TGCCGGCGTTTGTGCGAA-3’; mE1-E2, 5’-CACCTTTGGCAAATAAACGTGCGGCACGTTGTGATTAAGATCTAGCCGAT-3’ and 5’-TGCCGGCGTTTGTGCGAA-3’; E1-mE2, 5’-CACCTTTGGCAAATAAGATCTCGGAGATTTGTGATTACACGTGAGCCGAT-3’ and 5’-TGCCGGCGTTTGTGCGAA-3’; mE1-mE2, 5’-CACCTTTGGCAAATAAGATCTCGGAGATTTGTGATTAAGATCTAGCCGAT-3’ and 5’-TGCCGGCGTTTGTGCGAA-3’. The PCR products were inserted into the pENTR/D-TOPO vector using pENTR Directional TOPO cloning kit (Invitrogen), and then subcloned into the pHPdesteGFP vector, which expresses Green Fluorescent Protein (GFP) according to the enhancer sequence inserted [28], using Gateway LR-Clonase System (Invitrogen). The nucleotide sequences of all transgenes were confirmed by sequencing. The resulting transgenes were injected into embryos (BestGene) for recombination into the attp18 genomic site via PhiC31-mediated transgenesis to yield *tim* circadian enhancer GFP (*tim*-CEG) flies [52–54].

### Western blotting and protein quantification

Flies were entrained in a 12-h light/12-h dark (LD) incubator for at least 3 days, collected at the indicated time points, and frozen. Isolated frozen fly heads were homogenized in radioimmunoprecipitation assay (RIPA) buffer (20 mM Tris at pH 7.5, 150 mM NaCl, 1 mM EDTA, 0.05 mM EGTA, 10% glycerol, 1% Triton X-100, 0.4% sodium deoxycholate) containing 0.5 mM PMSF (phenylmethylsulfonyl fluoride), 10 μg/ml aprotinin, 10 μg/ml leupeptin, 2 μg/ml pepstatin A, 1 mM Na_3_VO_4_, and 1 mM NaF. This homogenate was sonicated 3 to 5 times for 10 s each time, using a Misonix XL2000 model sonicator at a setting of 3 and then centrifuged at 20,000 g for 10 min. The supernatant was collected as RIPA S extract, and protein concentration was determined by the Bradford assay. Equal amounts of RIPA S extract were run, transferred, and probed with guinea pig anti-CWO (GP-27), 1:5,000 and mouse anti-beta-actin (Abcom), 1:20,000. Horseradish peroxidase-conjugated secondary antibodies (Sigma) against guinea pig and mouse were diluted 1:5,000. Immunoblots were visualized using ECL plus (GE) reagent. Protein levels were measured by placing a rectangle of the same size over each CWO, ß-Actin or non-specific (NS) protein band on films used to visualize the immunoblots, and quantifying the signal within each rectangle via densitometric analysis using the ImageJ program. The levels of CWO were calculated as a CWO:ß-Actin or CWO:NS ratio, and CWO abundance at each time point was plotted relative to wild-type at ZT2.

### Chromatin Immunoprecipitation

Chromatin IP (ChIP) assays and qPCR quantification were performed as previously described [55]. CLK and CWO binding to E-boxes in the circadian enhancers upstream of *tim*, *per*, *vri*, and *Pdp1* in wild-type flies and the circadian enhancer in *tim*-CEG flies were first quantified via qPCR, and the resulting values were corrected for nonspecific binding to an intergenic region on chromosome 3R (nucleotides 29576172 to 29576303). The primers used for qPCR were as follows: for *tim* E-boxes, 5’-ACACTGACCGAAACACCCACTC-3’ and 5’-GCGGCACGTTGTGATTACACG-3’; for *per* E-boxes, 5’-GGGTGAGTAATGCCGTTGCGAAAT-3’ and 5’-ATTTGCTGGCCAAGTCACGCAGTT-3’; for *vri* E-boxes, 5’-CTGGTGCCTCACATTCCACG-3’ and 5’- CAGCAGTCAAGTTATAGCAGCGC-3’; for *Pdp1* E-boxes, 5’-GCACTCTCATTCTCTCTGTCGC-3’ and 5’-ACTTGGGGGACTGGAACTG-3’; for *tim*-CEG, 5’-GCCCCCTTCACCTTTGGCAAATA-3’ and 5’-TACAAGAAAGCTGGGTCGGCG-3’; and for the intergenic region, 5’-CAGGAGTCGVAGGACCAACC-3’ and 5’-GTCCTGAGAGGCTGAGAGGC-3’. PCR amplification using each pair of primers produced a single band of the expected size. The *tim*-CEG primers target vector sequences that flank the genomic *tim* E-box insert, and thus do not amplify endogenous *tim* genomic sequences.

### Quantitative RT-PCR

Quantitative RT-PCR was performed as described [55,56], with some modifications, to measure *GFP* mRNA levels. Total RNA was isolated from frozen fly heads using Trizol (Invitrogen), and treated with a Turbo DNase DNA-free kit (Ambion) to eliminate genomic DNA contamination. DNA-free total RNA (1.0 μg) was reverse transcribed using oligo(dT) 12–28 primers (Invitrogen) and Superscript II (Invitrogen). The reverse transcription (RT) product was amplified with SsoFast qPCR Supermix (Bio-Rad) in a Bio-Rad CFX96 Real-Time PCR System using primers to *GFP* (5’-TACGGCAAGCTGACCCTGAAGT-3’ and 5’-CGCACCATCTTCTTCAAGGACG-3’) and *ribosomal protein 49* (*rp49*) (5’-TACAGGCCCAAGATCGTGAA-3’ and 5’-GCACTCTGTTGTCGATACCC-3’). For each sample, mRNA quantity was determined using the standard curve for each gene analyzed. To determine the relative levels of GFP mRNA over a diurnal cycle, *GFP* mRNA levels were divided by *rp49* mRNA levels for each time point and plotted as the *GFP*/*rp49* mRNA ratio. To quantify *GFP* mRNA in different *tim*-CEG strains at the wild-type (E1-E2) peak, *GFP*/*rp49* values were normalized to the E1-E2 value at ZT14.

## Supporting Information

S1 Fig *tim* promoter fragments bearing E-box mutations abolish mRNA cycling. **(A)** Quantitative PCR (qPCR) was performed to measure GFP mRNA levels in E1-E2 *tim* circadian enhancer flies collected at the indicated times in LD. Relative GFP mRNA values were generated by dividing the GFP mRNA signal by that of ribosomal protein 49 (RP49), which is expressed at constant levels. Error bars represent the SEM (n = 3). **(B)** Quantification of GFP mRNA levels in E1-E2, mE1-E2, E1-mE2 and mE1-mE2 *tim* circadian enhancer transgenic flies collected at ZT14 as described in panel A. Relative GFP mRNA levels were normalized to the E1-E2 *tim* circadian enhancer fly value, which was designated as 1.0, then the means of each data set were calculated and plotted. Error bars represent the SEM (n = 3).
(TIF)
