# Supplementary figures and images for: CLOCKWORK ORANGE Enhances PERIOD Mediated Rhythms in Transcriptional Repression by Antagonizing E-box Binding by CLOCK-CYCLE
Source: PLoS Genet. 2016 Nov 4;12(11):e1006430. doi: 10.1371/journal.pgen.1006430 (PMC5096704; doi:10.1371/journal.pgen.1006430)

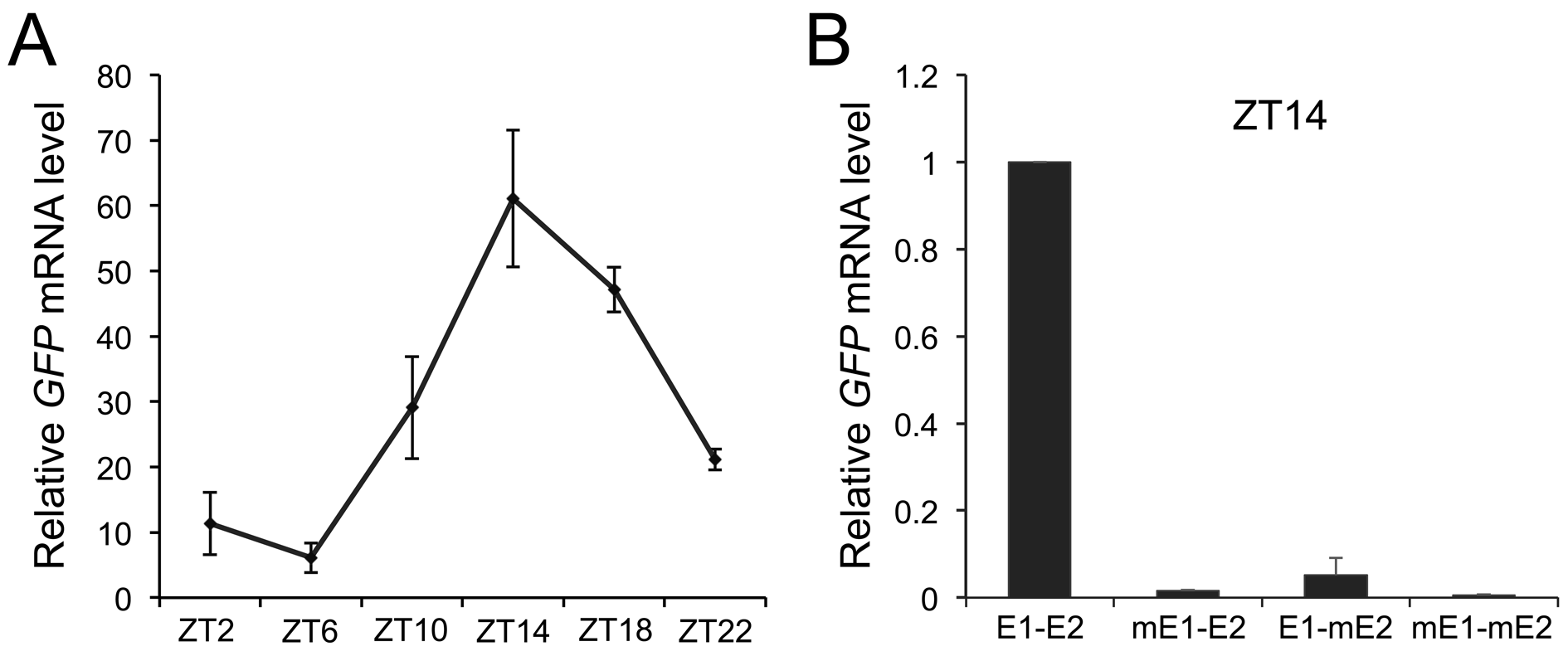

Supplement: S1 Fig — (A) Quantitative PCR (qPCR) was performed to measure GFP mRNA levels in E1-E2 tim circadian enhancer flies collected at the indicated times in LD. Relative GFP mRNA values were generated by dividing the GFP mRNA signal by that of ribosomal protein 49 (RP49), which is expressed at constant levels. Error bars represent the SEM (n = 3). (B) Quantification of GFP mRNA levels in E1-E2, mE1-E2, E1-mE2 and mE1-mE2 tim circadian enhancer transgenic flies collected at ZT14 as described in panel A. Relative GFP mRNA levels were normalized to the E1-E2 tim circadian enhancer fly value, which was designated as 1.0, then the means of each data set were calculated and plotted. Error bars represent the SEM (n = 3). (TIF) [file pgen.1006430.s001.tif]
